# Antiretroviral Drug Resistance Mutations among HIV Treatment Failure Patients in Tehran, Iran

**Published:** 2017-09

**Authors:** Molood FARROKHI, Samaneh MOALLEMI, Reza SHIRKOOHI, Reza GOLMOHAMMADI, Sara AHSANI-NASAB, Sara SARDASHTI, Ladan ABBASIAN, Kazem BAESI, Minoo MOHRAZ

**Affiliations:** 1. Iranian Research Center for HIV/AIDS, Iranian Institute for Reduction of High Risk Behaviors, Tehran University of Medical Sciences, Tehran, Iran; 2. Group of Genetics, Cancer Research Center, Cancer Institute of Iran, Tehran University of Medical Sciences, Tehran, Iran; 3. Dep. of Microbiology, Golestan University of Medical Sciences, Gorgan, Iran; 4. Hepatitis and AIDS Department, Pasteur Institute of Iran, Tehran, Iran

**Keywords:** Treatment failure, Drug resistance, Phylogeny, HIV

## Abstract

**Background:**

This study aimed to determine drug resistance mutations in patients with virological failure and find correlation between HIV drug resistance test and viral load.

**Methods:**

Blood sample was collected from 51 patients who suspicious treatment failure in the center of Imam Khomeini Hospital, Tehran, Iran in 2015. Viral voluntary counseling and testing load test was done and the patients with viral load above 1000 copies choose for detection of drug resistance mutations by genotyping method (29 patients).

**Results:**

The majority of patients (82.75) harbored the HIV subtype CRF 35 A-D. The 86.2% patients compromised at least one resistance mutation. The analysis of reverse transcriptase showed M184V (68.9%), T215YISF (44.8%), K103N (27.6%) and the analysis results of protease revealed G73SC (13.8%) and I47VA (6.9%). Eventually, the significant correlation between viral load and drug resistance was found.

**Conclusion:**

The result of our research stress the significance of recognizing drug resistant on time that prohibits the accumulation of drug resistance mutation and circulates the resistance strain of HIV-1 virus and the importance of national study according to the reliable findings for treatment guidelines.

## Introduction

Antiretroviral therapy (ART) has significantly diminished morbidity and mortality in patients with HIV. ART toxicity, pretreatment viral drug resistance, patients’ non-adherence to ART and hence inadequate suppression of viral replication and emergence of drug-resistant viruses are among the major causes of failure in treatment (1, 2). Viral replication under suboptimal antiretroviral pressure is due to accumulation of resistance mutations, which limit future therapeutic choices (3, 4). Drug adherence, a crucial factor in determining treatment outcome, may be tougher for patients with advanced disease (5). Non- adherence to therapy could result in suboptimal drug concentrations, therefore allowing viral replication to develop in the presence of the drug. This circumstance causes an ideal chance for selective drug resistance (6). Since evaluating the resistance profile of endemic subtypes yields valuable information for development of standard treatment protocols (4–6).

Drug resistance assessment can provide helpful information to clinicians whether to switch ARV regimens when treatment failure is suspected.

Growing access to first-generation drug combinations has led to concerns with regards to drug-resistance profiles of the patients living in low and middle-income countries (7, 8). However, the technology and assays are still very expensive and drug-resistance monitoring is available only on a limited basis (9). Moreover, “monitoring of clinical isolates for HIV DRMs (drug resistance mutations) is critical not only for the management of patients but also for policy makers forecasting drug needs following initial treatment failure” (10).

Standard first-line regimen in Iran is comprised of two nucleoside reverse transcriptase inhibitors (NRTIs) and one non-NRTI (NNRTI); (11) where medications are provided on a free-of-charge basis, and treatment initiation and monitoring have been guided by clinical and/or immunological data were available (12).

We aimed to perform drug resistance and HIV genotype testing in patients with failure in treatment. We also tried to assess possible correlations between demographic and laboratory measures in the context of drug resistance.

## Materials and Methods

### Study design, selection criteria, and definition of treatment failure

This case series study performed at Virology Laboratory of Iranian Research Center for HIV and AIDS (IRCHA). The laboratory works in close collaboration with a referral HIV Clinic at tertiary Imam Khomeini Hospital in Tehran in 2015, Iran. Patients were referred who were on ART treatment above 12 months and immunologically suspicious of treatment failure. Among them, 29 patients had viral loads above 1000 copies and were selected for drug resistance testing according to several references (13). Immunological failure was defined based on the WHO guidelines, consisting of the following criteria: decline of the CD4+ T-cell counts to baseline values (or below them); or 50% decline from on-treatment peak value; or CD4+ T-cell levels persistently below 100 cells/mm^3^ without concomitant infections (14).

### Ethical considerations

The study protocol was approved by the Institutional Review Boards (IRB) of Tehran University of Medical Sciences. The study was in line with the Declaration of Helsinki. All participants provided written informed consent after thorough explanation of the aims and objectives of the study. Considering blood sampling as a minimally invasive procedure, consent forms included a separate part of blood sampling. Participants were reassured about confidentiality of their medical records and test results; besides, we approved of their right to discontinue participation at any time during the study course upon their will.

### General measures

We gathered data regarding the general and demographic characteristics of the participants from their medical records (including age, route of transmission, duration of ART initiation, CD4+ T-cell counts, and time since first diagnosis confirmed by Western Blot test results).

### Primer design

In the initial step, the primer was designed utilizing Oligo Primer Analysis Software ver. 7 with HXB2 sequence and the best region was selected based on the prevalence among Iranian sequences (CRF 35AD, B, A1, C). Then to check for the specificity of the primer sequences, we re-checked them with the ones registered at the Basic Local Alignment Search Tool accessed through the page: http://blast.ncbi.nlm.nih.gov/Blast.cgi.

### Drug Resistance Testing

The viral RNA genome was extracted from a 200-μl sample of plasma with the QIAamp Viral RNA mini kit. Two sets of primers, consisting outer and inner primers were designed by Oligo 7 Software from the reference sequence with accession number NC-001802.1 available in NCBI website and were optimized for obtaining protease region sequence. The outer protease primers bound to nucleotides located between 1532 and 2207 (PR F1: RCA CMT AGC CAG RAA TTG following web C and PR R1: CTT TTA TTT TTT CTT CTG TCA ATG GCC) and inner primers bound to nucleotides between 1676 and 2166 (PR F2: TTT YCY TCA GAR CAG ACC AGA G and PR R2: TTC CTT CCT TTT CCA TTT CTG TAC A). Primers for reverse transcriptase gene used according to ANRS AC11 Resistance Study Group (15). The Outer reverse transcriptase primers of ANRS group bound to the nucleotides between 1685 and 2714(MJ3: AGTAGGACCTACACCTGTCA and MJ4: CTGTTAGTGCTTTGGTTCCTCT) and inner primers bound to nucleotides between 1735 and 2505(A35: TTGGTTGCACTTTAAATTTTCCCATTAGTCCTATT and NE1(35): CCTACTAACTTCTGTATGTCATTGACAGTCCAGCT). The complementary DNA was synthesized by QIAGEN One Step RT-PCR Kit (Qiagen, Hilden, Germany) and nested PCR for pro-tease and reverse transcriptase was performed with PCR materials from Fermentas Company. We also purified the PCR products using the QIAquick Gel Extraction Kit (Qiagen, Hilden, Germany). We complemented the sequencing of purified PCR products utilizing the Sanger method with forwarding inner primer for each gene (PR R2 and NE1) and evaluated the final products for drug resistance mutations and phylogenic analyses.

### Viral Load

Plasma viral load was quantified with the StepOne™ Real-Time PCR System by Real Star® HIV RT-PCR Kit (Altona, Hamburg, Germany) according to the manufacturer’s instructions.

### Drug resistance analyses

Drug resistance was evaluated by sequencing reverse transcriptase and protease genes amplified through the aforementioned methods. To check for type of resistant mutation and level of resistance to different ART classes, we obtained data through the Stanford HIV Drug Resistance Database (http://hivdb.stanford.edu/) (2, 16).

### Gene accession numbers

All of the sequences obtained in this study were submitted to Gen Bank database and are available under accession numbers KT793951 to KT793953, KT793955, KT793956, KT793960 to KT793971, KT793973, KT793975 to KT793977, KT793981, and KT793982. KT793993, KT793995 to KT793997 and KT7939400.

### Phylogenic analyses

All of the sequences obtained in the study used for phylogenic trees were accessed through MEGA 4 Software with neighbor joining method. To determine the subtypes, pol region sequences were aligned against the subtype and CRF reference sequences retrieved from the Los Alamos HIV-1 database (http://www.hiv.lanl.gov/). Final analyses were confirmed by the Comet online database (https://comet.lih.lu/) and the best Phylogenic trees were chosen that belonged to protease.

### Statistical Analysis

The statistical analysis was conducted using the SPSS ver.16.0 package (Inc. Chicago, IL, USA). Descriptive analyses were performed by calculating mean and standard deviation, median and interquartile range, as well as frequency. The non-parametric Mann-Whitney U test was used to compare the viral load in patients with and without drug resistance.

## Results

Data were analyzed for 29 participants. The mean age of the participants was calculated to be 32.21 yr and over half of the patients (n=16) were male and the mean time of HARRT use was 47.69 months. The median of viral load among them was 11000 and 11(37.9%) patients also were infected with HCV and HBV. Other general features are shown in Table 1.

**Table 1:** General and demographic characteristics of the study population among 29 patient with virological failure

**General/demographic variable**	**Value**
**Age** (yr); Mean (SD)	32.21(15.03)
**Gender**; Number (%)	
Male	16(55.2)
Female	13(44.8)
**Transmission route**; Number (%)	
Injection drug use	8(27.6)
Sexual	7(24.1)
Mother to child	7(24.1)
Blood products	3(10.13)
Unknown	4(13.8)
**Co-infection**; Number (%)	
Yes	11(37.9)
No	18(62.1)
**Viral load** (copies/μl); Median (IQR)	11000 (6925 – 130830)
**Time since diagnosis** (months); Mean(SD)	69.17(56.09)
**Duration of HAART use** (months); mean (SD)	47.69(30.31)
**Current treatment regimen**; Number (%)	
Standard Regimen	19 (65.5)
PI-based Regimen	10 (34.5)

### Drug resistance

From all samples (29 patients), 13.8% (n=4) of sequences from protease and reverse transcriptase did not show any drug resistance mutation. In the remaining 25 samples (86.2%), drug resistance mutations for the 3 classes of ART (NNRTI, NRTI, and PI) were observed and 4 samples showed resistance to all of them.

The most common mutation seen for the NRTIs was M184V and for the NNRTIs was K103N. For PIs, G73SC and I47VA were observed as the most common minor and major mutations, respectively. Details of the frequencies of various drug resistance mutations are indicated in Table 2. Based on the Stanford HIV Drug Resistance Database, the 25 patients with drug resistant viruses demonstrated different levels of resistance to 11 antiretroviral drugs.

**Table 2:** Frequency of drug resistance mutations for sequences obtained from the patient population

**Class of Medication**	**NRTI mutations**	**n (%)**	**NNRTI mutations**	**n (%)**	**PI mutations**	**n (%)**
**Type of Mutation**	M41L	4(13.8)	K101E	2(6.9)	**MINOR**	
	D67N	2(6.9)	K103N	8(27.6)	D30EF	3(10.34)
	K70ER	3(10.34)	E138AG	3(10.34)	G73SC	4(13.8)
	V75ML	6(20.7)	V179F	1(3.45)	**MAJOR**	
	M184V	20(68.9)	Y181C	6(20.7)		
	L210W	3(10.34)	Y188L	1(3.45)	I47VA	2(6.9)
	T215YISF	13(44.8)	G190SAT	6(20.7)	M46I	1(3.45)
	K219EQ	4(13.8)	F227L	1(3.45)	L90M	1(3.45)

The highest frequency of high-level drug resistance was demonstrated to be for lamivudine (72.4%) and emtricitabine (69%), while intermediate-level resistance showed to be the highest for abacavir (48.3%), stavudine (27.6%) and didanosine (27.6%). More information regarding the level of resistance to other ART medications is shown in Table 3.

**Table 3:** The level of resistance to 11 antiretroviral drugs for individuals with resistant strains

**Variable**	**High-Level n (%)**	**Intermediate-Level n (%)**	**Low-Level n (%)**
**NRTIs**			
3TC	21(72.4)		1(3.4)
FTC	20(69)		
AZT	7(24.1)	6(20.7)	3(10.3)
D4T	8(27.6)	8(27.6)	4(13.8)
DDI	2(6.9)	8(27.6)	10(34)
ABC	2(6.9)	14(48.3)	4(13.8)
TDF	1(3.4)	1(3.4)	9(31)
**NNRTIs**			
EFV	10(34)	6(20.7)	4(13.8)
NVP	15(51.7)	2(6.9)	3(10.3)
ETR	8(27.6)		7(24.1)
RPV	2(6.9)	6(20.7)	7(24.1)
**PIs**			
LPV	1(3.4)		2(6.9)
SQV	1(3.4)		3(10.3)
NFV	1(3.4)	2(6.9)	4(13.8)
ATV	1(3.4)		5(17.2)
DRV		1(3.4)	1(3.4)
FPV	1(3.4)	2(6.9)	1(3.4)
TPV			4(13.8)

ART, antiretroviral therapy; NNRTI, non-nucleoside reverse transcriptase inhibitor; NRTI, nucleoside/nucleotide reverse transcriptase inhibitor; PI, protease inhibitor; 3TC Lamivudine; FTC, emtricitabine; AZT, zidovudine; D4T, stavudine; DDI, didanosine; ABC, abacavir; TDF, tenofovir; EFV, efavirenz; NVP, nevirapine; ETR, etravirine; LPV, lopinavir; SQV, saquinavir; IDV, indinavir; NFV, nelfinavir; ATV, atazanavir; DRV, darunavir; FPV, fosamprenavir; TPV, tipranavir.

### Non-parametric analyses

Pursuant to Mann Whitney test the median of viral load was different between patients with and without drug resistance (U=91.5, P=0.004). The median of viral load for patients with drug resistance (median=29000, IQR=8936–262125) was more than patients without drug resistance (median=4225, IQR= 1525–8012).

### Phylogenic evaluation

**Fig. 1:**
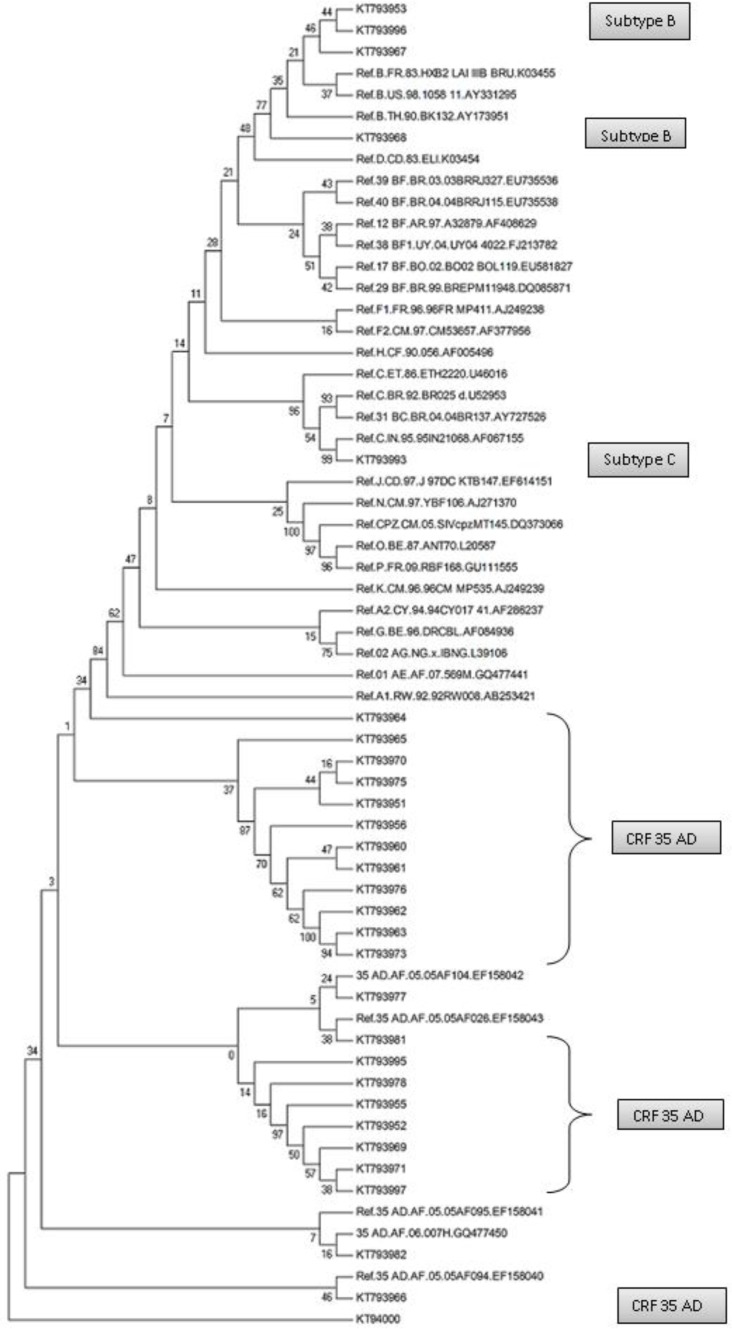
Constructed using MEGA4 software with neighbor joining method with 1000 replicates. The sequences with KT letter were result of whole length of protease sequences of the study (490 bp). Iranian subtype sequence was showed in vtext box

The complete region of Protease sequences (514 bp) used for Phylogenic analysis and most common subtype observed among the study population was CRF 35AD 82.75(n=24). Four patients had subtype B (13.8%) and only one patient had subtype C (3.45%) (Fig. 1).

## Discussion

In the present study, we evaluated drug resistance and phylogenetic profile of patients with treatment failure. Among 29 patients, 86.2% of the patients showed some level of drug resistance.

This has been the first time that drug resistance has been investigated among patients referred through clinicians with suspected clinical or immunological failure in our region. Although high level of drug resistance was revealed in another study in Iran, our sample comprised of ambulatory patients. Half of the participants who enrolled in the study were women, probably reflecting indicating the increase in number of women infected with by HIV in the country (17).

The frequency of drug resistance in our study shows more patients with drug resistance compared with the results of similar studies performed in the Ethiopia, Morocco and Saudi Arabia. In the Ethiopia due to measurement of viral load per 6 months and rapid finding of drug resistance mutation in patients, the number of patients with drug resistance was low (18).

Results of drug resistance studies in Morocco represents that 53% of the sequences were exhibition of at least one DRM (19) and Saudi Arabia indicate 41% resistance in patients (20). The findings of our studying are explained to be related to non-adherence to ART or late recognition of treatment failure (21).

The frequency of M184V mutation, as the major cause of high-level drug resistance to NRTIs, was more commonly observed than other mutations similar to the findings of other studies (19, 22). This mutation is related to development of resistance to lamivudine that is part of the standard of care provided in Iran. This mutation also leads to cross-resistance to emtricitabine whereas the mutation increases the sensitivity of viruses to Zidovudine, Stavudine, and Tenofovir (5).

For NNRTIs the most common mutated sequence was reported to be K103N that is similar to the findings of other studies (23). This mutation is known to limit Nevirapine and Efavirenz drug efficacy. In addition, the low genetic barrier of the NNRTI drugs causes resistances to these drugs (24).

On the other side, G73SC and I47VA were common mutations seen for PIs. Unlike the other classes of ART drugs, the profile of drug resistance to PIs in the present study shows differences compared to other studies (25, 26). G73SC mutation is associated with reduced susceptibility to nelfinavir and saquinavir. I47VA confers high-level resistance to lopinavir and fosamprenavir; as well low/intermediate-resistance to the remaining PIs expect for atazanaavir and saquinavir (27). Kaletra (lopinavir/ritronavir) was the main PI prescribed to patients in our country; hence, we predict treatment failure to be seen among patients with I47VA mutation. This finding is controversial to studies that performed drug resistance analysis among patients receiving lopinavir including the Moroccan study (19). Hence, we recommend more studies to investigate the distribution of I47VA mutation at national level to help with development of treatment guidelines.

The median of viral load was different between patients with and without drug resistance. In addition, the median viral load ≥ 4225 copy per ml were closely correlated with the development of drug resistance similar to another study performed in Ethiopia and China (18, 28). This suggested that HIVDR was one of the most important factors associated with virological failure. Subtype CRF 35AD predominated among participants similar to the previous study (29–32). The CRF 35AD subtype may relate to the CRF35AD Afghanistan (33). The relation is due to the annual immigration of Afghan refuge to Iran (34).

This study has some limitations. Drug resistance test was not carried out before the patients initiating ART initiation and also measuring adherence to treatment was not established except through patient files, so we cannot claim all the resistance mutations occur after initiation of ART or because of non-adherence to ART cause resistance mutation.

## Conclusion

This study provides an update on the molecular epidemiology of HIV-1 in patients with virological failure in a referral center. The findings highlight the importance of timely recognition of treatment failure to prevent accumulation of resistance mutations and transition of the resistance viruses HIV-1 strain in population as well as the need for national-level studies to base treatment guidelines on reliable evidence. To provide clinicians with simple and affordable viral load tools remains another challenge to be tackled by policymakers in the region.

## Ethical considerations

Ethical issues (Including plagiarism, informed consent, misconduct, data fabrication and/or falsification, double publication and/or submission, redundancy, etc.) have been completely observed by the authors.
